# Prevalence of Hepatitis D Virus Infection Among Patients With Chronic Hepatitis B Attending Birjand Hepatitis Clinic (East of Iran) in 2012

**DOI:** 10.5812/hepatmon.11168

**Published:** 2013-08-25

**Authors:** Masood Ziaee, Ghodsieh Azarkar

**Affiliations:** 1 Hepatitis Research Center, Department of Internal Medicine, Birjand University of Medical Sciences, Birjand, IR Iran; 2 Department of Radiology, Birjand University of Medical Sciences, Birjand, IR Iran

**Keywords:** Delta Hepatitis, Chronic Hepatitis B, Seroprevalence

## Abstract

**Background:**

Hepatitis delta virus (HDV) is a defective RNA virus dependent on Hepatitis B virus (HBV) infection for its replication and expression. All patients with HBV infection should be tested for the presence of HDV infection. It is estimated that approximately 5% of hepatitis B surface antigen (HbsAg) carriers in the world are HDV infected patients. HBV-HDV co-infection may lead to more severe acute disease and higher risks of fulminant hepatitis, cirrhosis, and hepatocellular carcinoma than those having HBV infection alone. Also, HBV infected patients with HDV super-infection have a higher rate of progression to chronic disease and serious complications.

**Objectives:**

Our aim was to determine the prevalence of HDV infection among chronic hepatitis B (CHB) patients attending Birjand Hepatitis Clinic, East of Iran.

**Materials and Methods:**

A cross-sectional analytical study was conducted on 413 CHB patients in 2012. Serology test for anti-HDV was measured by ELISA in these patients. CHB patients had positive hepatitis B surface antigen for at least 6 months before the study entrance.

**Results:**

The mean age of CHB patients was 38.5± 11.9 years and 55.9% of them (231 patients) were male. There were 13 cases (3.1%) with HDV infection. There was no association between positive anti-HDV serology and factors such as age, gender, carrier state, liver enzymes, and positive hepatitis B e antigen (HBeAg) serology.

**Conclusions:**

Although HDV had a low prevalence in our area, it is important for healthcare providers and policy makers to plan preventive strategies for HDV spread as well as HBV prevention programs among high risk population.

## 1. Background

Hepatitis B virus (HBV) infection is a worldwide distributed diseasewith a high rate of mortality and morbidity; infecting about 2000 million people of which 350 to 400 million have chronic hepatitis B (CHB) infection. It is estimated that 65 million CHB patients will die from liver disease due to their HBV infection (1, 2).

CHB is a condition in which the hepatitis B surface antigen (HBsAg) persisted for 6 month from its first detection. The possibility of developing CHB varies with the age of the patient, and its risk decreases from about 90% in neonates whose mothers are positive for hepatitis hepatitis B e antigen (HBeAg) to 5% in adults (3, 4).

All patients with HBV infection should be evaluated for the presence of co-infection with other viral infections including hepatitis C, hepatitis D, and human immunodeficiency virus (HIV). These co-infections result in a poorer prognosis than HBV infection alone (5).

Hepatitis delta virus (HDV) is a defective RNA virus dependent on HBV infection for its replication and expression (6). In 1977, it was first described by Rizzetto et al (7) as a co-infection or super-infection in HBV infected patients. It is estimated that more than 15 million people have HDV infection all across the world. However, its prevalence is higher in Italy, Eastern Europe, and Western Asia, and it is endemic in the Middle East (8). HDV with a universal distribution has a rate of approximately 5% among hepatitis B surface antigen (HbsAg) carriers worldwide (9). The routes of HDV transmission are similar to those for HBV, including blood-borne, sexual, percutaneous, permucosal, and perinatal transmission. However, patients having HBV-HDV co-infection may have more severe acute disease and higher risks of fulminant hepatitis, cirrhosis, and hepatocellular carcinoma (HCC) than those having HBV infection alone (10). Also, HBV infected patients with HDV superinfection have a higher rate of progression to chronic disease and serious complications (11).

## 2. Objectives

The aim of this study was to determine the prevalence of HDV infection among CHB patients attending Birjand Hepatitis Clinic, in East of Iran.

## 3. Materials and Methods

This cross-sectional analytical study was conducted on CHB patients in 2012. The study protocol was approved by the ethics committee of Birjand University of Medical Sciences and all study participants gave written informed consent before being included in the study. To calculate the sample size, N = z^2^ p (1-p)/d^2^ formula was used; where N was the minimum required sample size; Z score was 1.96 for the confidence level 95%; p, the proportion affected; and d, the desired precision of this expected proportion. The approximate prevalence of the problem was assumed 6.61% (0.0661) (12) and its desired precision (d) was 0.03, then the minimum sample size was calculated 264. So, all patients (n=312) who had referred to the investigator in Hepatitis Clinic between 2002 and 2012, Birjand, Iran, with a history of chronic HBV infection, were called to participate in the study. Patients who had incomplete information on the study questions were excluded from the analysis (n=5). During the study period, 106 new cases of CHB were added to the study sample, and totally 413 CHB patients were included for the analysis through a consecutive sampling of eligible patients (i.e. it was a convenience sample). The confidentiality of the patient’s data was ensured by the lack of any identifying personal information. The data were gathered by either patients direct questioning or results of laboratory testing, and were recorded into an information form including demographic characteristics, risk factors, and laboratory tests. The risk factors included history of blood transfusion, intravenous drug abuse, tattooing, cupping, surgery, endoscopy, and hemodialysis. The laboratory tests consisted of HBV serum markers, HBsAg, anti-hepatitis B core, hepatitis B e antigen (HBeAg), anti-HBeAg, and anti-hepatitis D virus (anti-HDV), and were determined by commercial immunoenzymatic assays (DIA PRO Diagnostic Bioprobes, Srl., Italy). Chronic hepatitis B was defined as having positive hepatitis B surface antigen for at least 6 months before the study entrance. The inactive HBsAg carrier state was defined by absence of HBeAg and presence of anti-HBe along with repeatedly normal serum alanine aminotransferase (ALT) levels (13). An abnormal ALT and Aspartate Aminotransferase (AST) level was defined as values greater than 40 IU.L^-1^.

Values are presented as means± standard deviation (SD) for quantitative variables and frequency (percentage) for qualitative variables. The values were compared between anti-HDV positive and negative patients using the independent t-test or Mann–Whitney test. A chi-square or Fisher’s exact test was used to compare categorical variables. Logistic regression analysis was performed to calculate the odds ratio (OR) and 95% confidence intervals (CI). Statistical analysis was carried out using (SPSS, Chicago, Illinois, USA, and version 17) statistical software and the statistical significant level were defined as a P value less than 0.05.

## 4. Results

Totally, 413 chronic HBsAg positive patients were included in the study. Their main characteristics are presented in Table 1. The mean age of patients was 38.5±11.9 (ranged between 7 and 77) years. Study included 231 (55.9%) males (mean age 39.1 ±11.5 years) and 182 (44.1%) females (mean age 37.7 ± 12.4 years). Of the total participants, 343 (83.1%) were married, 235 (59%) living in the city, 132 (32.2%) housewives, and 93 (22.7%) in private business. A history of HBV infection in family members was seen in 163 (39.5%) patients. HBeAg was detected in the serum of 59 (14.3%) patients, and 343 (83.1%) patients were seropositive for anti-HBe. The number of subjects with HDV infection was 13 (3.1%), and one patient had HCV infection. 

**Table 1.** General Characteristics of Chronic Hepatitis B Patients

	Study patients			
	All (n = 413)	HBeAg positive (n = 59)	HBeAg negative (n = 353)	P value
**Age, y, Mean ± SD (range) **	38.5 ± 11.9 (7-77)	34.6 ± 12.6 (9-70)	39.1 ± 11.7 (7-77)	0.008
**Gender, No. (%)**				0.575
Male	231 (55.9)	35 (59.3)	195 (55.2)	
Female	182 (44.1)	24 (40.7)	158 (44.8)	
**Marital status, No. (%)**				0.014
Single	70 (16.9)	17 (28.8)	53 (15.0)	
Married	343 (83.1)	42 (71.2)	300 (85.0)	
**Living status, No. (%)**				0.794
Rural area	147 (36.9)	17 (32.7)	129 (37.4)	
Urban area	235 (59)	33 (63.5)	202 (58.6)	
Abroad	16 (4)	2 (3.8)	14 (4.1)	
**Occupation, No. (%)**				0.004
Housewives	132 (32.2)	15 (25.9)	117 (33.3)	
Private business	93 (22.7)	21 (36.2)	72 (20.5)	
Employee	69 (16.8)	3 (5.2)	65 (18.5)	
Student	35 (8.5)	10 (17.2)	25 (7.1)	
**Military occupation**	30 (7.3)	2 (3.4)	28 (8)	
Retired	11 (2.7)	2 (3.4)	9 (2.6)	
Other	40 (9.7)	5 (8.6)	35 (10)	
**HBV in family, No. (%)**	163 (39.5)	39 (66.1)	123 (34.8)	< 0.001
**Some risk factors, No. (%)**				
Tattooing	7 (1.7)	-	7 (2)	0.600
Cupping	37 (9)	3 (5.1)	34 (9.6)	0.331
Surgery	81 (19.6)	8 (13.6)	73 (20.7)	0.287
Endoscopy	57 (13.8)	4 (6.8)	53 (15)	0.104
**HBeAg positive, No. (%)**	59 (14.3)	NA	NA	
**Anti-HBe positive, No. (%)**	343 (83.1)	NA	NA	
**Anti-HDV positive, No. (%)**	13 (3.1)	3 (5.1)	10 (2.8)	0.411
**Abnormal ALT, No. (%)**	138 (34.8)	31 (55.4)	107 (31.5)	0.001
**Abnormal AST, No. (%)**	107 (27.2)	28 (50)	79 (23.4)	< 0.001

Two groups were formed on the basis of HDV status; 13 patients whose anti-HDV test was positive were allocated to HDV positive group, and 400 patients who tested negative to anti-HDV were assigned to HDV negative group. Comparative analysis of HDV positive group and HDV negative group showed no statistical difference in age, the male: female ratio, living area, occupation, frequency of risk factors, presence of HBV infection in family members, and abnormality in laboratory tests. (Table 2) 

**Table 2.** Comparison Between Anti-Hepatitis D Virus Positive and Negative Groups for Demographics, Risk Factors, HBeAg Serology, and Liver Enzymes

	Study patients		
	Anti-HDV positive(n = 13)	Anti-HDV negative(n = 400)	P value
**Age, y, Mean ± SD **	38.2 ± 11.6	38.5 ± 11.9	0.915
**Gender, male, No. (%)**	7 (53.8)	224 (56)	> 0.999
**Marital status, Married, No. (%)**	12 (92.3)	331 (82.8)	0.705
**HBV in family, No. (%)**	6 (46.2)	157 (39.2)	0.774
**Tattooing**	-	7 (1.8)	> 0.999
**Cupping, No. (%)**	1 (7.7)	36 (9)	> 0.999
**Surgery, No. (%)**	1 (7.7)	20 (80)	0.487
**Endoscopy, No. (%)**	3 (23.1)	54 (13.5)	0.402
**HBeAg positive, No. (%)**	3 (23.1)	56 (14)	0.411
**Anti-HBe positive, No. (%)**	9 (69.2)	334 (83.5)	0.248
**Serum ALT (IU.L), Mean ± SD** ^**-1**^ **), Mean ± SD**	39.68 ± 38.61	44.15 ± 28.26	0.231
**Serum AST (IU.L), Mean ± SD** ^**-1**^ **), Mean ± SD**	33.71 ± 29.86	32.15 ± 11.68	0.430

No difference was seen in the HBeAg status in relation to gender, living situation, risk factors, HDV infection, and abnormality in some laboratory tests. However, HBeAg-positive patients were more likely to be younger (age, 34.6 vs. 39.1 years; P = 0.008), to be single (24.3% vs. 12.3%, P = 0.014; OR = 2.29, 95% CI: 1.21-4.32), to have activities of non-governmental (and non-military) organizations (17% vs. 5.1%, P = 0.002; OR=3.82, 95% CI: 1.84-9.85), to have HBV infection in family members (24.1% vs. 8%, P < 0.001; OR = 3.64, 95% CI: 2.04-6.52), to have an abnormal rise in ALT levels (22.5% vs. 9.7%, P = 0.001; OR = 2.7, 95% CI: 1.52-4.79), and also to have a rise in AST levels (26.2% vs. 9.8%, P < 0.001; OR = 3.27, 95% CI: 1.83-5.84).

## 5. Discussion

In this study we found that the rate of HDV infection among CHB patients is 3.1%. This rate is similar to other Iranian studies which have reported the prevalence of HDV in Iranian HBV infected population. However, a wide range of prevalence rates between 2% and 17.3% was observed in these Iranian studies. In center of Iran, the rate of HDV was 2% in Qom province (14) and 2.9% in Isfahan (15). It was 5.8% in Golestan Province (in the North)(16-18), 9.3% in Tabriz and Tehran Hepatitis Clinics (in the North-West and central area) (19), and 17.3% in Hamedan Province (in the West) (20). The prevalence of HBV in different areas as well as the sampling methods may influence the results in different Iranian studies, where the study in Hamedan whose HDV rate was the highest among other Iranian studies included a high number of intravenous drug abuse subjects in the study (20). The variations in HDV prevalence may be due to factors that influence the HDV transmission such as the generally lower socioeconomic status in some areas. Amini et al in a meta-analysis of Iranian studies published in 2011 (12), estimated that the HDV prevalence in Iran was 6.61%. However, as a country of the Middle East, Iran is located in endemic area for HDV (8) and it may require more efficient concentrated screening, prevention, and public education programs. This may be more important when some stated that the rate of HDV co-infection has been increasing during the past decade in the country (20). In the Middle East, the prevalence of HDV infection among asymptomatic carriers of HBsAg in Jordan, Saudi Arabia, Turkey, and Kuwait was reported 2%, 3.3%, 5.2%, and 31%, respectively (9). HDV is endemic in Tajikistan and Pakistan. In Pakistan, our neighboring country, HDV is highly endemic and had rates between 16.6% and 88.8% in reports from different areas(21). It is a major medical problem in southern and eastern Turkey and an intermediate to high endemic infection in Eastern European countries (21). Outbreaks of HDV between 1980 and 1990 were reported from Samara (Russia), Greenland, and Mongolia (21). In Europe, the prevalence of HDV was reported less than 5% in Austria, Ireland, France, Poland, Belgium, Croatia, Bulgaria, and Switzerland (21). Although, the rate of HDV infection in a study from Germany was 11%, only 20% of participants were originated in Germany and the others were immigrants from Turkey or Eastern Europe (21). The rate of 8.5% for anti-HDV was reported in England and 85% of them were from Southern or Eastern Europe, sub-Saharan Africa, or Asia (21). A similar scenario has been found in some other European countries, e.g. France and Greece. A 6.3% prevalence of HDV was seen in Northern California, USA(21).

Our study found no difference between anti-HDV positive and negative patients regarding age, gender, risk factors, and other laboratory tests. Similarly, in North Iran, Roshandel et al (17) found no significant relationship between anti-HDV seropositivity and demographic factors such as age, place of residence, and marital status. In contrast, Somi et al (19) in Tabriz, stated that HDV was significantly higher after reaching 40 years of age, but not statistically different between men and women. for younger than 30’s and the 30’s, 40’s,50’s, and 60’s and older than the 60’s ,respectively. However, in a linear model for logistic regression, a history of dental treatment, and several trips abroad were associated with HDV infection and other HBS risk factors including surgical interventions, blood transfusion, needle sticks, tattooing, cupping, extramarital sexual contacts, intravenous drug abuse, family history of hepatitis, place of residence (rural/urban), and war injuries were not good predictors for HDV infection (19). Bakhshipour et al (22) reported the rate of 17% for HDV among HBV-infected patients in the city of Zahedan, the capital of the province of Sistan and Balouchestan (southern neighbor of our province). Although, positive family history, tattooing, cupping, and dental manipulation were major risk factors for HBV among their patients, many of the patients did not have an identifiable risk factor for HDV, and they concluded that probably unsafe injection in the past was responsible for infection in their patients. In contrast to ours and other Iranian studies, they found a different HDV risk between both genders as 0.36 times less in women and concluded that it may be due to greater rate of high risk behaviors in men (22). Similar to the study of Zahedan, in Pakistan (eastern neighbor of Sistan and Balouchestan province), Khan et al (23) reported a rate of 28% for HDV and indicated a significantly higher rate of HDV among male patients, but regarding to age, no significant difference was seen in patients with ages above or below 40 years. Another study from Pakistan (24) found that 31.5% of their patients were anti-HDV positive and no significant difference was found between positive and negative groups regarding to age, gender, marital status, and place of residence. In a study by Celen et al (25) in Turkey, another neighboring country, anti-HDV was positive in 6% of asymptomatic HBV carriers and in 27.5% of active CHB patients. They demonstrated significant association between anti-HDV positivity and the duration of HBsAg carrier status, but no association with age, gender, and HBeAg positivity. In the Northern Africa, in Tunisia, a comparisiontooke place between 176 asymptomatic carriers originated from regions of variable HBV endemic cities and 39 CHB patients with HDV positive serology, and the results showed that the mean age of patients was 5 years higher in the HDV positive subjects than in the global population (26). Similarly, two African studies by Mansour et al (27, 28) in Mauritania, a high endemic area for HDV, found that HDV infection was seen more in older ages. They also reported that HDV was associated with male gender (28), number of marriages, military profession, residence in the desert, and a history of hospitalization (28). Two studies were performed in western Brazilian Amazon, another high prevalence area for HDV; in one of them, Viana et al (29) stated that HDV was associated with Amerindian ethnic origin, lower educational level, history of acute viral hepatitis, history of malaria, male gender, tattooing, and older age. Similarly, Braga et al (30) reported that HDV infection was significantly associated with age (15 years or older), the type of HBV infection among HBsAg carriers, and history of previous clinical hepatitis among patients in western Brazilian Amazon area. However, they found no association with gender, history of surgery, tattooing, use of illicit drugs, sharing a toothbrush, or previous hepatitis B vaccination. In multivariate analysis, HDV infection remained associated with the type of HBV infection, hepatitis history, and older ages. They concluded that the increased risk to older ages, especially between 20 to 39 years, may show the importance of sexual transmission in their area (30).

Also, the rate of HDV positivity in our study had no difference between patients with positive and negative HBeAg serology. Some authors indicated that HDV infected patients had a low level of HBV DNA in both HBeAg-negative and HBeAg-positive groups suggesting suppressive effects of HDV on HBV irrespective of the phase of HBV infection. Furthermore, they found that clinical long-term outcome was similar in both groups (31). As expected, patients with active HBV infection had higher rates of single status, HBV infection in family members, abnormal ALT/AST tests, and were less employed in governmental/military occupations. Active HBV infected patients may have more psychological distress, impairment in social activities, limitation in working capacity, or even restrictions on hiring them for certain jobs, which all may influence their life patterns such as marital,familial, and working status.They may infect their family members or they may even have been infected by close exposure to infected family members.

This study has some limitations, such as the method used for HDV detection. The method used for HDV detection was ELISA but the confirmation of ongoing HDV infection by PCR testing of HDV RNA was not performed in our study. Also, HBV DNA viral load by PCR was not measured in this study.

Although our study demonstrated that Birjand is an area with a low prevalence for HDV, it is important for healthcare providers and policy makers to plan preventive strategies for HDV spread as well as HBV prevention programs. Also, further studies are needed to investigate the risk factors associated with HBV and HDV infections and the background reasons for the regional increase in anti-HDV serology of HBV carriers.
